# A novel anoikis-related gene signature predicts prognosis in patients with breast cancer and reveals immune infiltration

**DOI:** 10.1097/MD.0000000000035732

**Published:** 2023-10-27

**Authors:** Chaoyi Tang, Liuqing Qin, Jiehua Li

**Affiliations:** a Department of Gastrointestinal Gland Surgery, The First Affiliated Hospital of Guangxi Medical University, Nanning, China.

**Keywords:** anoikis, breast cancer, prognosis, signature, tumor microenvironment

## Abstract

Breast cancer (BRCA) is a common malignancy worldwide that is associated with a high mortality rate. Despite recent improvements in diagnosis and treatment, there is an urgent need to investigate the processes underlying cancer progression and identify novel prognostic indicators. Anoikis, which plays a role in the development of human malignant tumors, has been gaining increasing interest from researchers. However, the potential role of anoikis-related genes (ANRGs) in the advancement of BRCA remains unknown. In this study, we aimed to assess the predictive value of ANRGs in BRCA, construct a prognostic model based on ANRGs, and explore the tumor microenvironment in different prognostic score groups. This study utilized data from the Cancer Genome Atlas (TCGA) and Gene Expression Omnibus (GEO) databases to collect clinical information and RNA sequencing data from patients with BRCA. Information on ANRGs was gathered from GeneCards and Harmonizome portals. A risk score model based on ANRGs was created using least absolute shrinkage and selection operator Cox (LASSO) regression analysis. Additionally, the study explored the tumor microenvironment and enriched pathways in different risk groups. Finally, a novel ANRG-based nomogram is developed. A total of 142 differentially expressed genes associated with survival were identified, of which 5 genes were selected to create the ANRG signature. The risk score based on this signature proved to be an independent prognostic factor. Further analysis revealed that different risk subgroups exhibited variations in the tumor microenvironment and drug sensitivities. Subsequently, a nomogram was developed using risk scores and clinicopathological factors. The decision curve analysis results suggest that patients with BRCA might derive clinical treatment benefits from utilizing this prognostic model. Based on the results of this study, the ANRG signature and nomograph established can be used for clinical decision-making in patients with BRCA.

## 1. Introduction

Breast cancer is the most common malignancy and second leading cause of death in women. It accounts for 31% of all incident cases and 15% of all deaths in females.^[1]^ The current treatment approach for breast cancer involves surgical resection combined with radiation therapy, anti-human epidermal growth factor receptor 2 (HER2) therapy, and endocrine therapy. The progression of tumor development from normal cells to cancer cells is influenced by the deterioration of the patient’s health and proliferation of tumorigenic factors. Throughout this process, cancer cells accumulate mutations and differentiate into different genetic lineages and subgroups, resulting in tumor heterogeneity.^[2]^ Tumor heterogeneity, which is closely linked to the invasive and metastatic potential of tumors, affects clinical diagnosis, treatment, and prognosis.^[3]^ Therefore, it is crucial to identify reliable and efficient clinical therapeutic techniques and biomarkers to improve the survival and cure rates of breast cancer patients.

Anoikis is a type of programmed cell death that occurs when cells lose contact with the extracellular matrix (ECM). Its main role in the human body is to prevent the abnormal attachment of detached tumor cells, which is crucial for maintaining homeostasis.^[4]^ Certain malignancies that increase the risk of distant metastasis are associated with tumor cells that have high resistance to anoikis. These cells can survive in the bloodstream and establish secondary tumors in distant locations.^[5–7]^

Anoikis-related genes have been found to play a significant role in the progression of various types of cancer, including ovarian cancer,^[8]^ osteosarcoma,^[9]^ gastric cancer,^[10]^ and breast cancer.^[11]^ One study found that BMP4 promotes resistance to anoikis and chemoresistance in MDA-MB-231 breast cancer cells by upregulating the NOTCH signaling pathway.^[12]^ Additionally, upregulation of the A2 thromboreagin (TP) receptor and the A2 synthetase 1 (TBXAS1) TP receptor has been observed in metastatic breast cancer cells. However, aspirin has been shown to promote anoikis and inhibit breast cancer metastasis through COX inactivation and to decrease TAX2 by generating anoikis resistance through sustained Akt activation and subsequent stimulation of breast cancer metastasis to the lung.^[13]^ Despite these findings, there has been limited exploration of breast cancer prognostic models based on anoikis-related genes (ANRGs).

This study aimed to examine the predictive value of ANRGs in breast cancer and develop a prognostic model based on ANRGs. Additionally, this study aimed to investigate the tumor microenvironment in different prognostic score groups.

## 2. Materials and methods

### 2.1. Acquisition of clinical data and gene expression analyses

Gene expression information of 1060 breast cancer tissues and 111 adjacent normal tissues was collected from The Cancer Genome Atlas (TCGA) database. Our analysis focused on 1042 breast cancer tissues with complete clinical information (Table S1, Supplemental Digital Content, http://links.lww.com/MD/K462). RNA sequencing expression data were normalized using the FPKM method. Additionally, we obtained the GSE48390 dataset from the Gene Expression Omnibus (GEO) database, which consisted of 80 breast cancer samples with accessible clinical data (Table S2, Supplemental Digital Content, http://links.lww.com/MD/K463). The data from this dataset were normalized to remove batch effects.

### 2.2. Acquisition of anoikis-related genes

A total of 520 ANRGs were downloaded from the GeneCards database and Harmonizome portal. ANRGs in the GeneCards database were selected based on a relevance score of > 0.4 as a screening requirement.

### 2.3. Clustering of different anoikis-related patterns

In the TCGA-BRCA dataset, we utilized the “limma” R package to assess the differentially expressed ANRGs (Anoikis-Related Genes) between breast cancer and normal tissues. To identify ANRGs associated with prognosis, we performed univariate Cox regression analysis and set the threshold at “*P* < .05.” Additionally, we employed the “ConsensusClusterPlus” R package to identify the clustering patterns related to anoikis. The expression patterns of 16 different ANRGs indicating prognosis were analyzed using k-means clustering. To ensure an optimal number of clusters, we utilized a consensus matrix and cumulative distribution function. Furthermore, we evaluated the stability of anoikis-associated patterns through principal component analysis (PCA), t-distributed stochastic neighbor embedding (t-SNE), and uniform manifold approximation and projection (UMAP).

### 2.4. Functional enrichment analysis

The gene set variation analysis (GSVA) R package was used to enrich the Kyoto Encyclopedia of Genes and Genomes pathway in various clusters. The c2.cp.KEGG.v7.4.symbols.gmt files were acquired from The Molecular Signatures Database. The gene set enrichment analysis (GSEA) was performed using the clusterprofiler package of R.

### 2.5. Construction and evaluation of an anoikis-related prognostic signature

To develop a prognostic signature, we employed LASSO regression and multivariable Cox regression analyses to identify the genes associated with prognosis. The risk scores were then characterized using tenfold cross-validation, and the λ values were determined based on the minimum partial likelihood of deviance. The ANRG-based risk score was calculated using the following formula: risk score = expr-gene1 × coefficient1 + expr-gene2 × coefficient2 + ... + exprgeneN × coefficientN. where coefficientN represents the risk coefficient, and expr-geneN represents the gene expression level for each gene. To assess the predictive ability of the model, we constructed Kaplan–Meier (KM) survival curves and performed time-dependent receiver operating characteristic (ROC) curve analyses.

### 2.6. The link between risk score and immune cell infiltration

Using CIBERSORT and estimate R scripts, we calculated the relative proportion of infiltrating immune cells. To identify the relationship between immune-infiltrating cells and the risk scores, we performed Spearman’s rank correlation analysis.

### 2.7. Establishment and validation of a predictive nomogram

A nomogram was developed using clinicopathological factors and risk scores. The accuracy was verified through internal validation using calibration charts. The net clinical benefit was evaluated using decision curve analysis (DCA).

### 2.8. Evaluation of drug sensitivity in the risk Subgroups

To examine the predictive ability of ANRG characteristics for chemotherapeutic drugs, we utilized the “oncoPredict” R package to compute the half-maximal inhibitory concentrations (IC50) of clinical drugs in various risk subgroups. Subsequently, we evaluated the results to determine drug sensitivity.

### 2.9. Tumor immune single-cell hub database and Kaplan–Meier plotter database

The Tumor Immune Single-cell Hub is an extensive online database containing single-cell RNA sequences. It provides a systematic exploration of tumor microenvironment heterogeneity across various datasets and cell types. The KM Plotter is another database that allows for the investigation of the correlation between gene expression and survival across 21 different tumor types. These databases source their data from GEO, European Genome-Phenome Archive, and TCGA.

### 2.10. Statistical analysis

Statistical analyses were performed using the v4.2.0 R software package. Differentially expressed genes were screened using a threshold of log2|fold change| > 1.0, *P* < .05, and a false discovery rate of < 0.05. Statistical significance was set at *P* < .05.

## 3. Results

### 3.1. Identification of prognosis-related ANRGs

**Figure 1.**
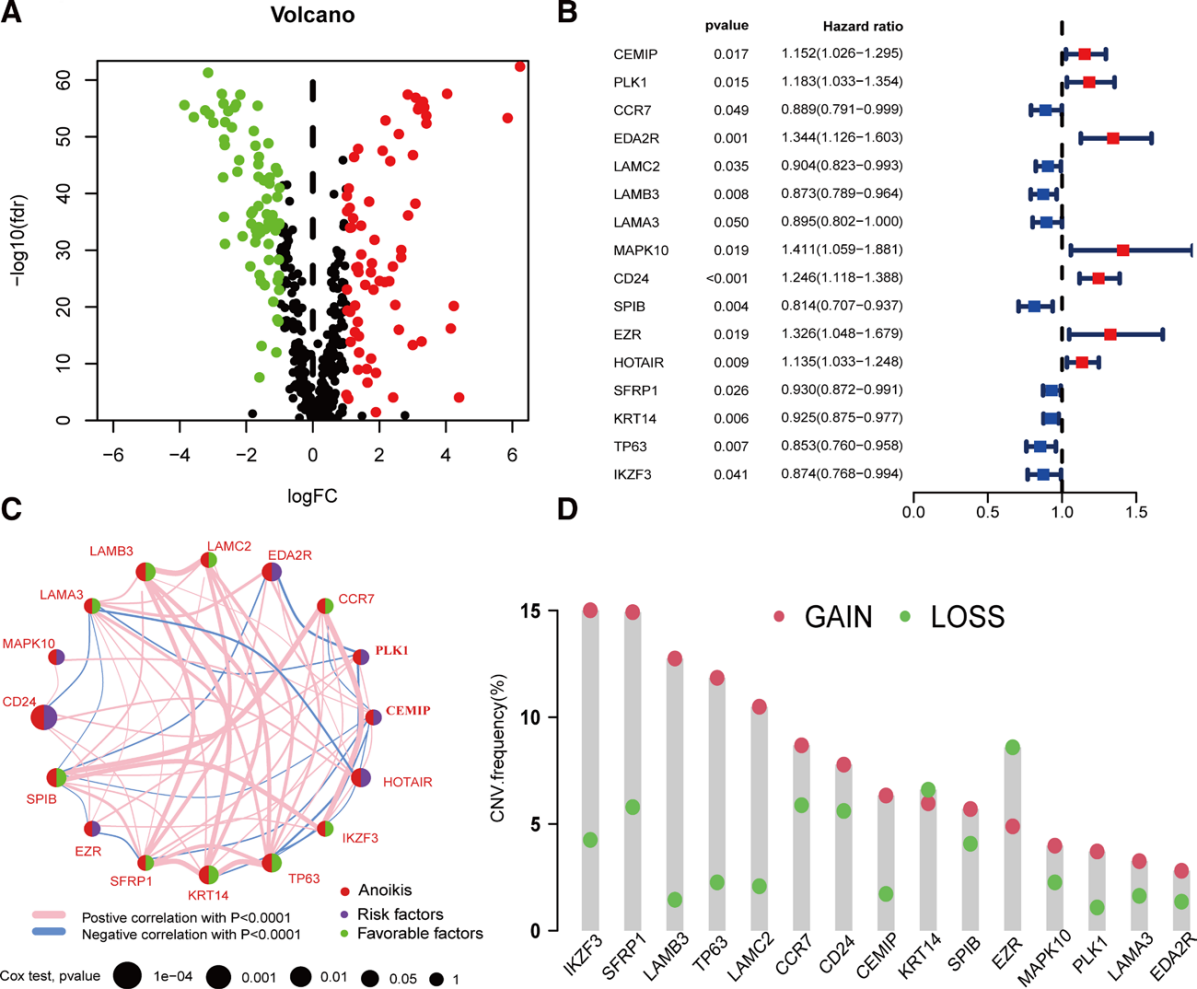
Features of ANRGs in breast cancer. (A) A total of 142 distinct ANRGs were identified in the TCGA_BRCA dataset. (B) Forest plot depicting the 16 ANRGs associated with prognosis. (C) Network diagram illustrating the associations among the 16 ANRGs associated with prognosis. (D) Copy number variations of ANRGs. ANRGs = anoikis-related genes, BRCA = breast cancer, TCGA = The Cancer Genome Atlas.

In total, 520 ANRGs were retrieved from GeneCards and Harmonizome. Among these, 142 ANRGs showed differential expression in breast cancer tissues compared to normal breast tissues in the TCGA_BRCA dataset (Fig. 1A). Further analysis using univariate Cox regression identified 16 ANRGs associated with prognosis, and forest plots were created to visualize the results (Fig. 1B). The relationship between the expression levels of all 16 ANRGs was further elucidated through a network plot. Notably, a significant positive correlation was observed between LAMC2 and LAMB3 (Fig. 1C). The genes LAMC2 and LAMB3 encode the laminin subunit gamma-2 and laminin subunit 3 proteins, respectively. These proteins are essential components of Laminin-332, a complex that plays a crucial role in cell adhesion, migration, and the maintenance of tissue integrity. Mutations in either of these genes have been associated with the development of Epidermolysis Bullosa, a group of inherited skin disorders characterized by skin fragility and blister formation.^[14]^ Copy number variation (CNV) data were acquired from the TCGA database and analyzed. A bar graph was generated to visualize the frequency of copy number changes for each gene. EZR and KRT14 were more likely to exhibit a decrease in copy number, whereas the remaining genes showed the opposite trend (Fig. 1D). Additionally, 3 genes, IKZF3, SFRP1, and LAMB3, exhibited the highest mutation levels.

### 3.2. Consistent clustering of breast cancer cohorts according to 16 ANRGs

**Figure 2.**
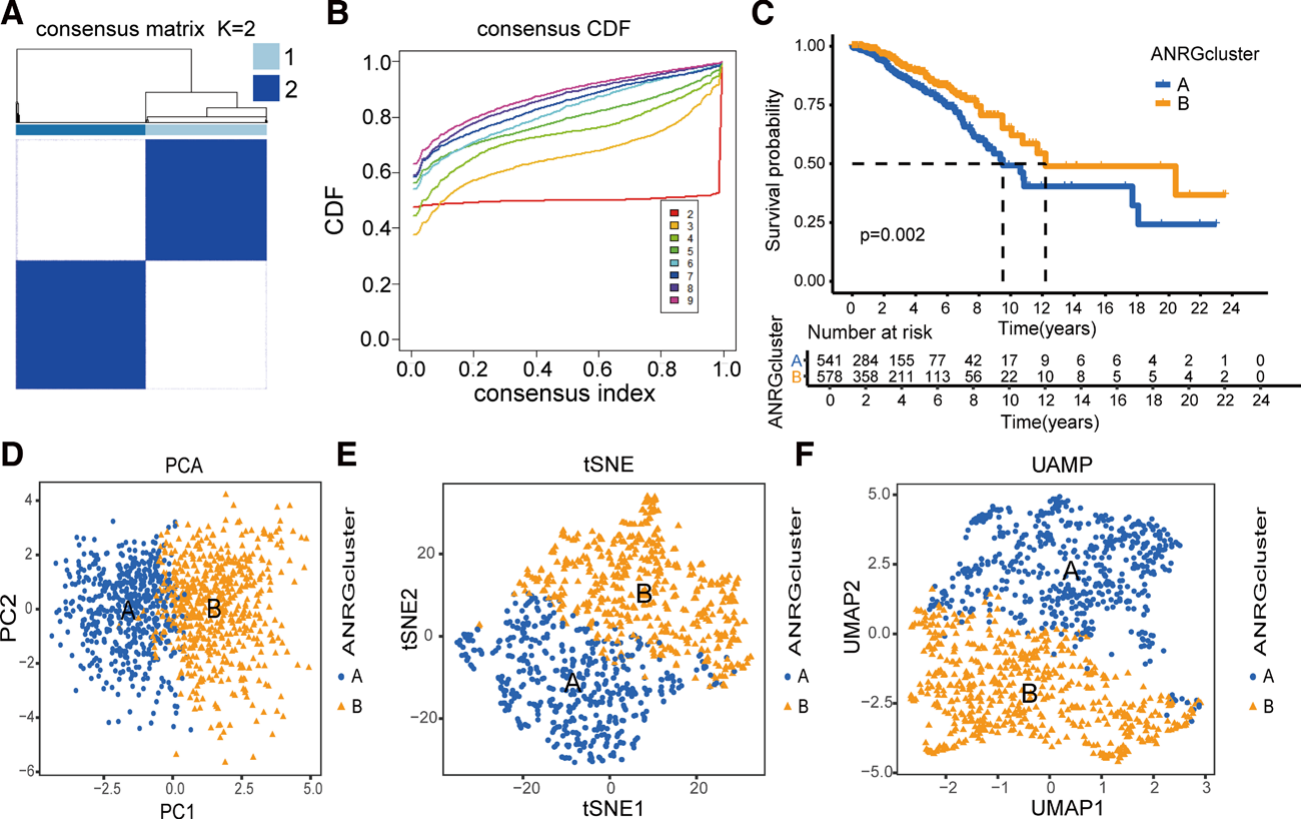
Consistent clustering based on expression profiles of ANRGs (A, B) Patients with breast cancer are classified into 2 clusters. (C) The prognosis of the 2 clusters shows significant differences. (D–F) Principal component analysis, t-distributed stochastic neighbor embedding, and uniform manifold approximation and projection analysis of patients with breast cancer between ANRG cluster A and B. ANRGs = anoikis-related genes.

To investigate the clinical significance of ANRGs in patients with breast cancer, we conducted consensus clustering using the Consensus Cluster Plus package in R. When k = 2, the cohorts were successfully classified into 2 distinct subtypes (Fig. 2A and B). KM survival curves revealed a significant difference in the outcomes between the 2 groups (*P* = .002; Fig. 2C). We further validated the accuracy of this clustering using PCA, UMAP, and t-SNE, which confirmed the clear identification of these 2 clusters (Fig. 2D–F).

### 3.3. Gene expression, immune infiltration, GSVA, and GSEA in the 2 subtype clusters

**Figure 3.**
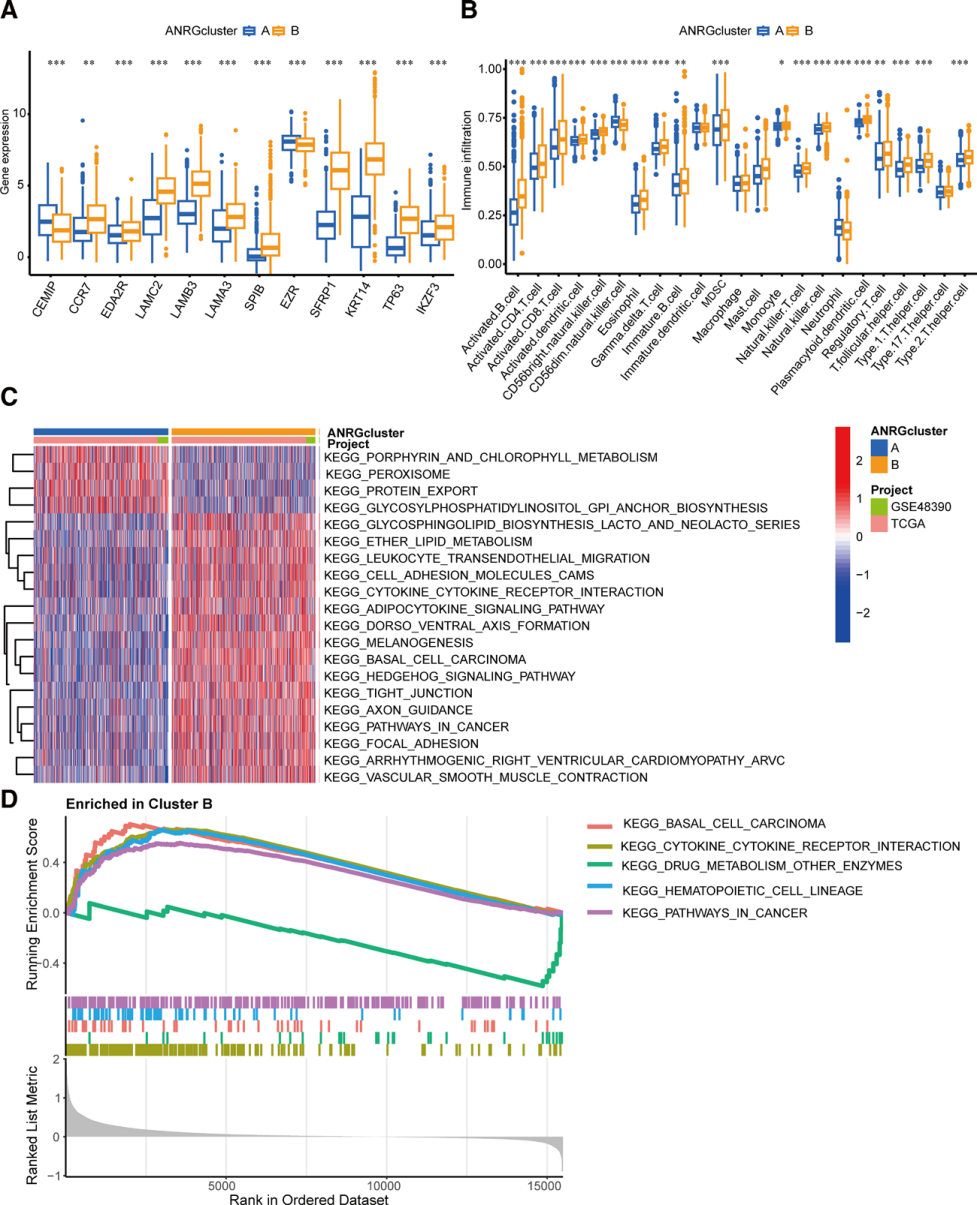
Gene expression, immune infiltration, GSVA, and GSEA in the 2 clusters. (A) Expression of ANRGs in the 2 clusters. (B) Immune infiltration patterns in the 2 clusters. (C, D) GSVA and GSEA focus on the enrichment of the Kyoto Encyclopedia of Genes and Genomes pathways between the 2 clusters. *P < .05, **P < .01, and ***P < .001. ANRGs = anoikis-related genes, GSEA = gene set enrichment analysis, GSVA = gene set variation analysis.

Boxplots revealed that ANRG expression patterns were present in both subgroups. In cluster B, the expression levels of CCR7, EDA2R, LAMC2, LAMB3, LAMA3, SPIB, SFRP1, KRT14, TP63, and IKZF3 were considerably higher than those in cluster A. However, the expression of the remaining genes showed contrasting results (Fig. 3A). The differentially expressed genes between the 2 clusters were associated with patient prognosis, making them potential targets for tumor therapy. Additionally, significant differences in immune cell infiltration were observed, with cluster B having a lower fraction of natural killer cell CD56dim than cluster A (Fig. 3B). Furthermore, pathways, such as basal cell carcinoma, cytokine-cytokine receptor interaction, and pathways in cancer, which were co-validated by GSVA and GSEA, were significantly enriched in cluster B. This cluster, associated with a worse prognosis, played a crucial role in promoting tumor proliferation and evasion of apoptosis (Fig. 3C and D).

### 3.4. Building and validating an anoikis-related prognostic signature

**Figure 4.**
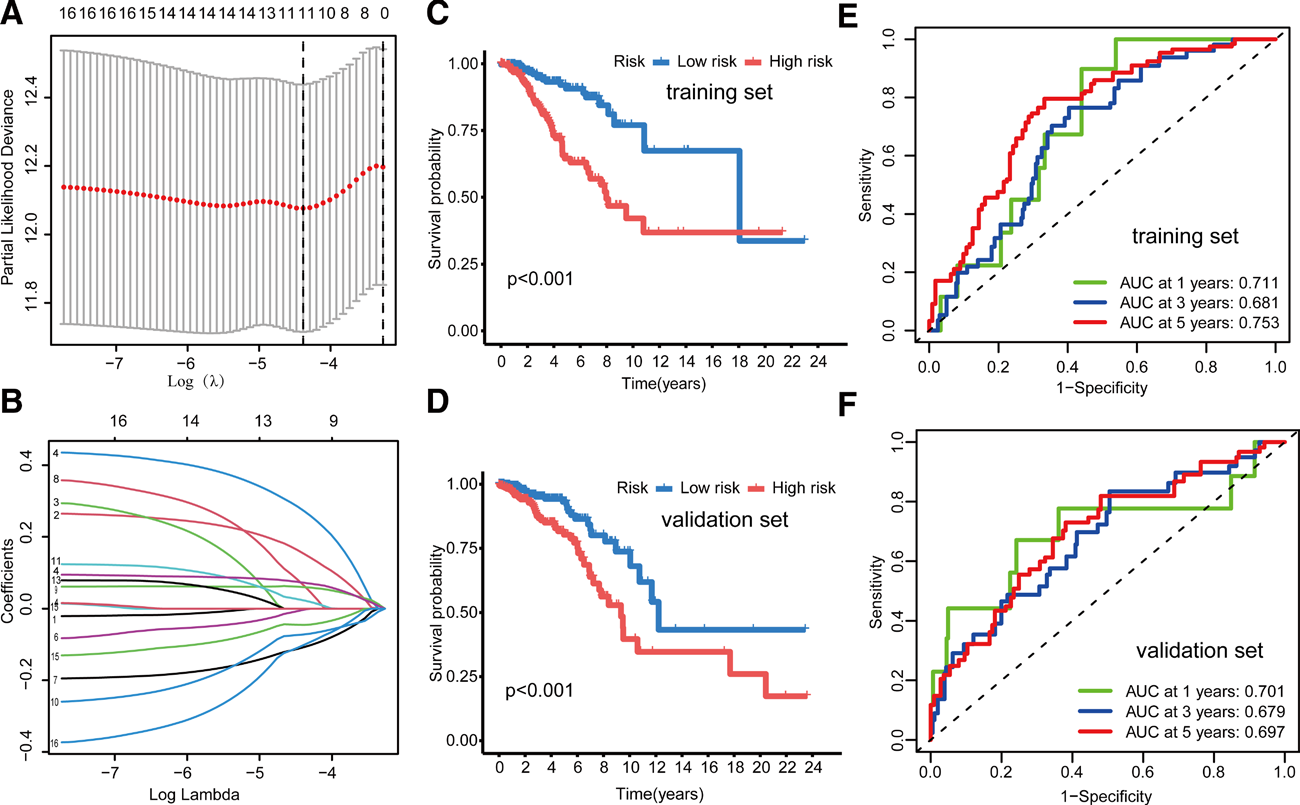
Construction and validation of the prognostic signature of anoikis-related genes (ANRGs). (A) Identification of the 5 genes that constitute the ANRG signature using the least absolute shrinkage and selection operator regression analysis. (B) Coefficient profile plots of the 5 ANRGs. (C, D) Kalan-Meier curves show a significant difference in overall survival between the 2 subgroups in the training and validation data sets. (E, F) Time-dependent receiver operating characteristic curves at 1, 3, and 5 years follow-up in the training and validation data sets.

To investigate the prognostic significance of the ANRGs, we performed LASSO regression analysis to exclude overfitting genes from a pool of 16 candidate genes. The ANRG signature, comprising 5 genes (PLK1, EDA2R, LAMA3, HOTAIR, and IKZF3), was constructed using multiple regression analysis (Fig. 4A and B). Risk scores based on ANRGs were calculated using the following formula: risk score = (PLK1 × (0.2957) + (EDA2R × (0.4616) + (LAMA3 × (−0.2065) + (HOTAIR × (0.1282) + (IKZF3 × (−0.2303). KM analysis also demonstrated that patients with high-risk breast cancer had a worse prognosis, which was confirmed in both the training and validation datasets (Fig. 4C and D). ROC curves were constructed to evaluate the ANRG signature validity. The results showed that the areas under the curve were 0.711, 0.681, and 0.753 for 1, 3, and 5 years, respectively, in the training set, and 0.701, 0.679, and 0.753697 for 1, 3, and 5 years, respectively, in the validation set (Fig. 4E and F).

### 3.5. Correlation between the tumor microenvironment and ANRG signature

**Figure 5.**
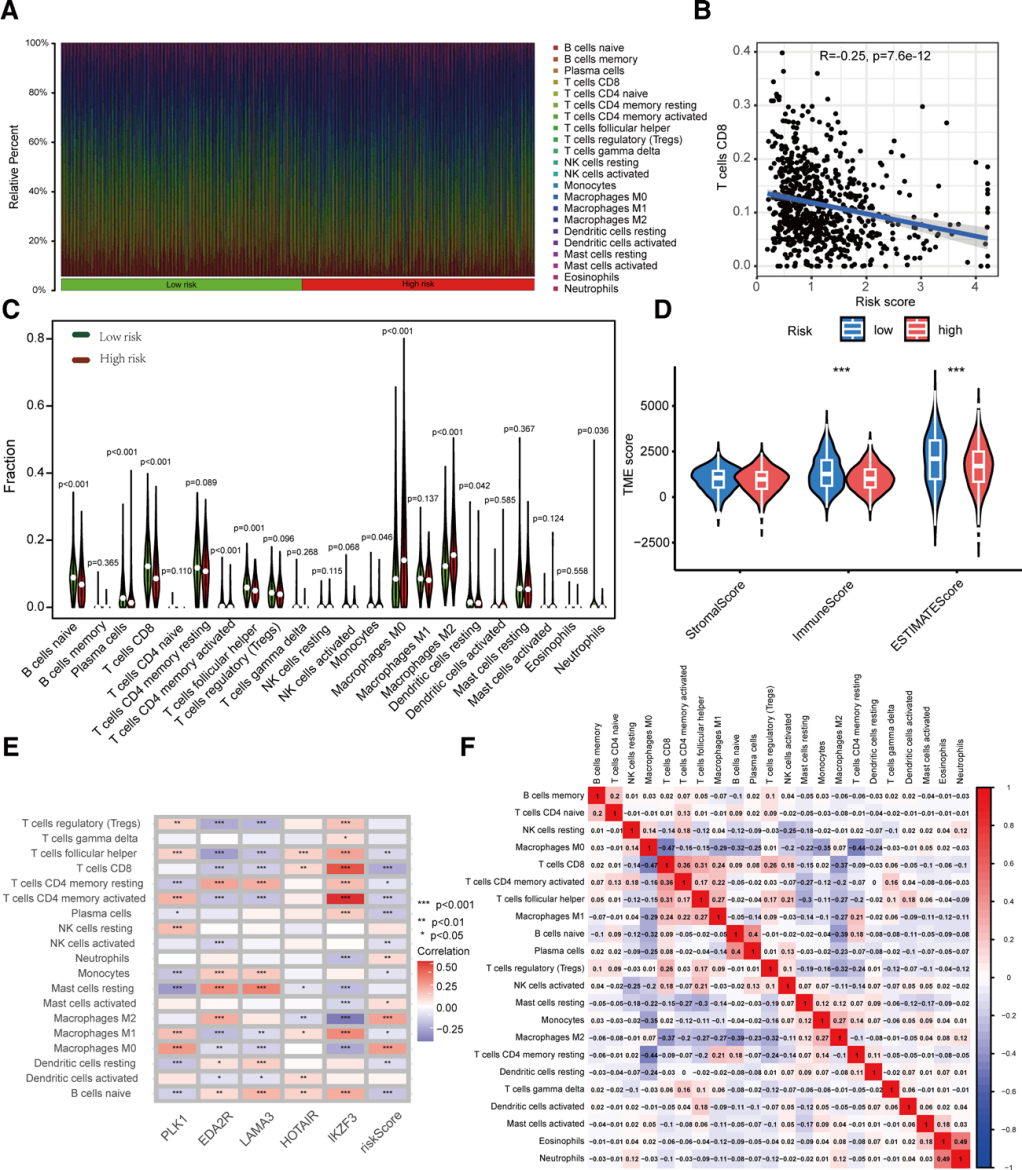
Correlation between tumor microenvironment and signature of anoikis-related genes (ANRGs). (A) The ratio of infiltrating immune cells in the high-risk group and the low-risk group. (B) Correlation between risk score and the proportion of CD8+ T cells. (C) Comparison of immune cell components between the 2 risk groups. (D) Comparison of estimate scores between the different risk groups. (E) Relationship between immune cells and the 5 ANRGs. (F) Correlation between immune cells.

The role of the tumor microenvironment in tumorigenesis and immunotherapy is of great importance. In this study, we investigated the landscape of the tumor microenvironment in patients with breast cancer. Using CIBERSORT, we estimated the proportion of immune cell types in both low- and high-risk groups. Patients with breast cancer were initially ranked based on their risk score, and it was observed that the proportion of different immune cells corresponded to the risk score (Fig. 5A). Specifically, there was a significant decrease in the proportion of CD8+ T cells as the risk score increased (*R* = −0.25, *P* = 7.6e–12; Fig. 5B). Furthermore, we found that the high-risk group had a significantly lower proportion of native B cells, plasma cells, and CD8+ T cells than the low-risk group. In contrast, the proportion of M0 and M2 macrophages was significantly higher in the high-risk group (Fig. 5C). Expression profiles were estimated to obtain matrix scores, immune scores, and estimation scores for the high- and low-risk groups of patients with breast cancer (Fig. 5D). The high-risk group had a higher proportion of tumor cells and a lower proportion of immune cells than the low-risk group. The heat map depicts the relationship between the proportion of infiltrating immune cells and the expression of the 5 genes. The expression of IKZF3 showed a remarkably positive relationship with the proportion of activated CD8+ T cells and CD4+ memory T cells, whereas it was negatively correlated with M2 macrophages (Fig. 5E). Pearson’s correlation heatmap revealed different correlations between different immune cells. There was a significant positive correlation between neutrophils and eosinophils and a significant negative correlation between M0 macrophages, activated CD8+ T cells, and CD4+ memory T cells (Fig. 5F).

### 3.6. Establishment of a prognostic nomogram for breast cancer

**Figure 6.**
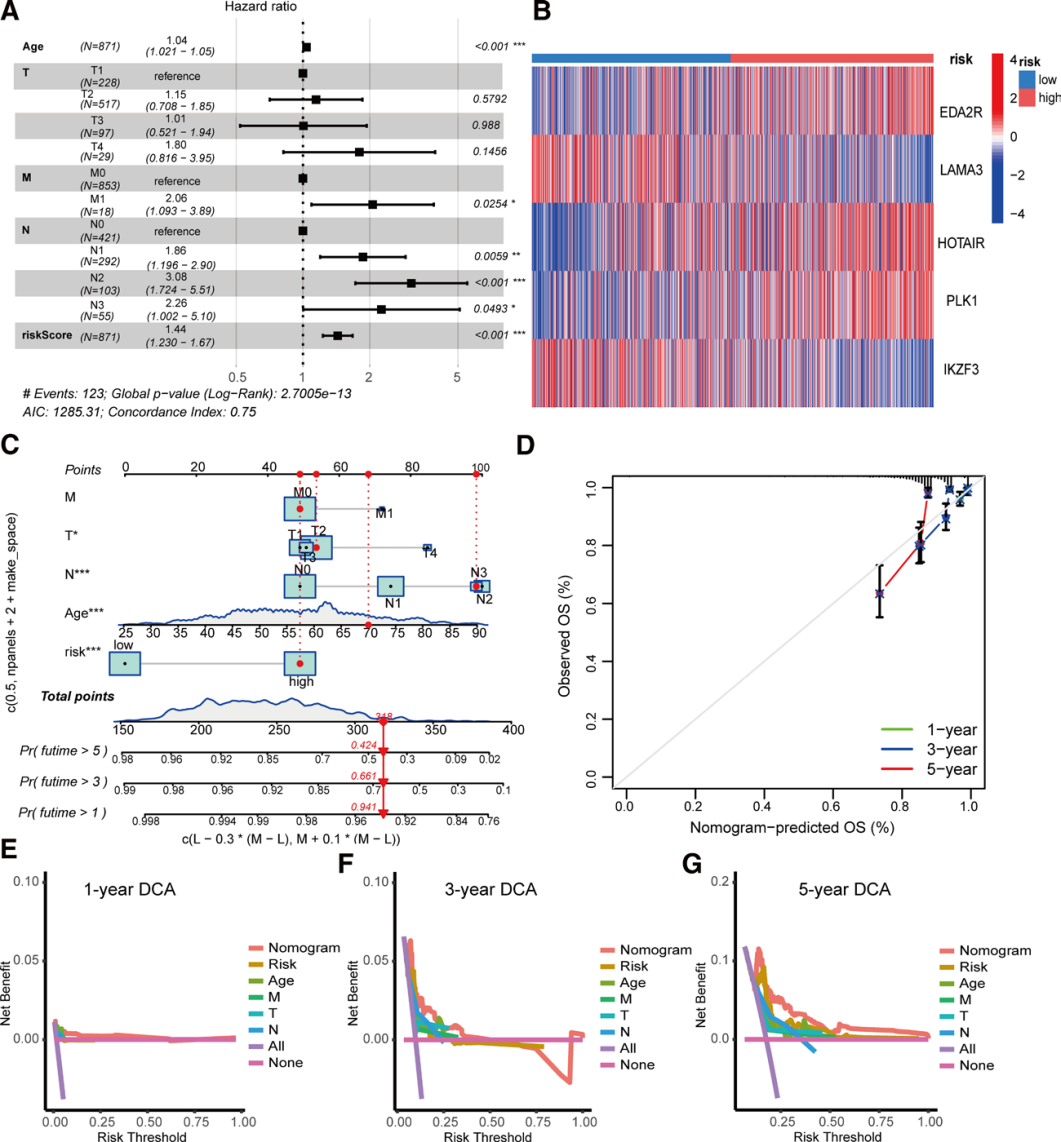
Construction and assessment of a prognostic model. (A) Multivariate Cox regression analysis of clinicopathological factors and risk score. (B) Relationship between risk score and expression of the 5 ANRGs. (C) Construction of the nomograph. (D) Calibration plots of the nomograph. (E-G) Decision curve analysis of the nomogram and clinicopathological factors for overall survival (OS) at 1, 3, and 5 years. ANRGs = anoikis-related genes.

To assess the prognostic potential of the ANRG signature, a multivariate Cox regression analysis was conducted considering the ANRG risk score and clinicopathological factors. The forest map (Fig. 6A) demonstrated that age, lymph node metastasis, and risk score were independent risk factors for survival in breast cancer. The heatmap (Fig. 6B) shows the correlation between the 5 ANRGs and risk score. A novel nomogram incorporating clinicopathological factors and the ANRG signature was developed to further enhance the prognostic value of the ANRG signature (Fig. 6C). Calibration curves indicated that this nomogram had a high prognostic predictive value (Fig. 6D). Decision curve analysis (DCA) is a commonly used approach to evaluate clinical prognostic models, diagnostic procedures, and molecular indicators. In this case, the new nomogram proved to be a valuable method for predicting outcomes in patients with breast cancer (Fig. 6E–G).

### 3.7. Relationship between drug sensitivity and risk score

**Figure 7.**
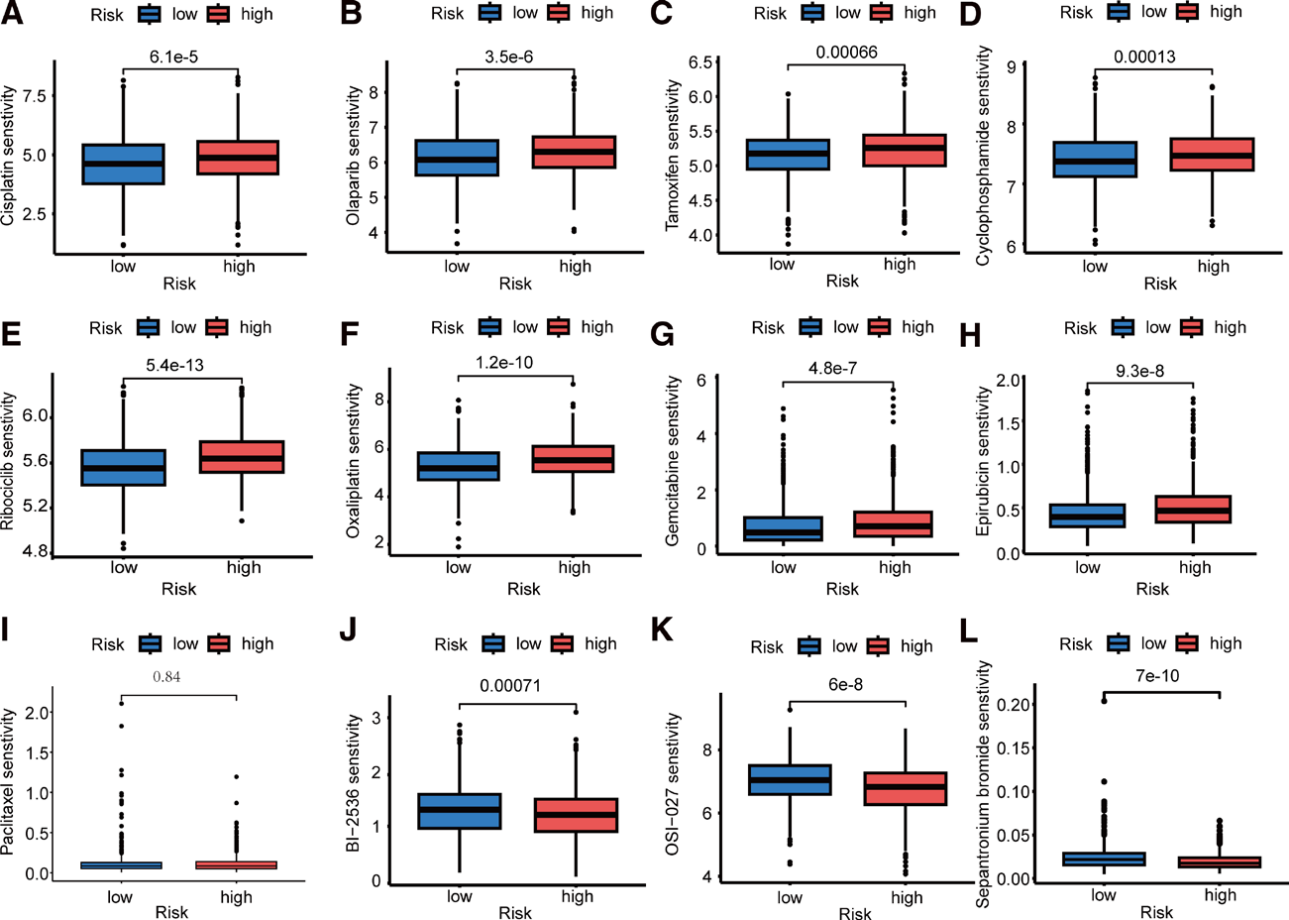
Drug sensitivity analysis. (A–L) Box plots showing the sensitivity of the 12 drugs (cisplatin, olaparib, tamoxifen, cyclophosphamide, ribociclib, oxaliplatin, gemcitabine, epirubicin, paclitaxel, BI-2536, and OSI-027, and sepantronium bromide) between the different risk groups.

In this study, we initially selected 9 chemotherapeutic agents commonly used for breast cancer treatment from the Genomics of Drug Sensitivity in Cancer (GDSC) database for drug sensitivity analysis. Among these agents, 8 (cisplatin, olaparib, tamoxifen, cyclophosphamide, ribociclib, oxaliplatin, gemcitabine, and epirubicin) showed low sensitivity in high-risk breast cancer cases (Fig. 7A–H), whereas one agent (paclitaxel) showed no difference in sensitivity between the risk subgroups (Fig. 7I). To identify drugs that exhibited relatively high sensitivity in the high-risk group, we further analyzed all drugs in the GDSC database and found 3 drugs (sepantronium bromide, BI-2536, and OSI-027) that demonstrated relatively high sensitivity in the high-risk group (Fig. 7J–L).

### 3.8. Correlation between ANRGs and the tumor microenvironment

**Figure 8.**
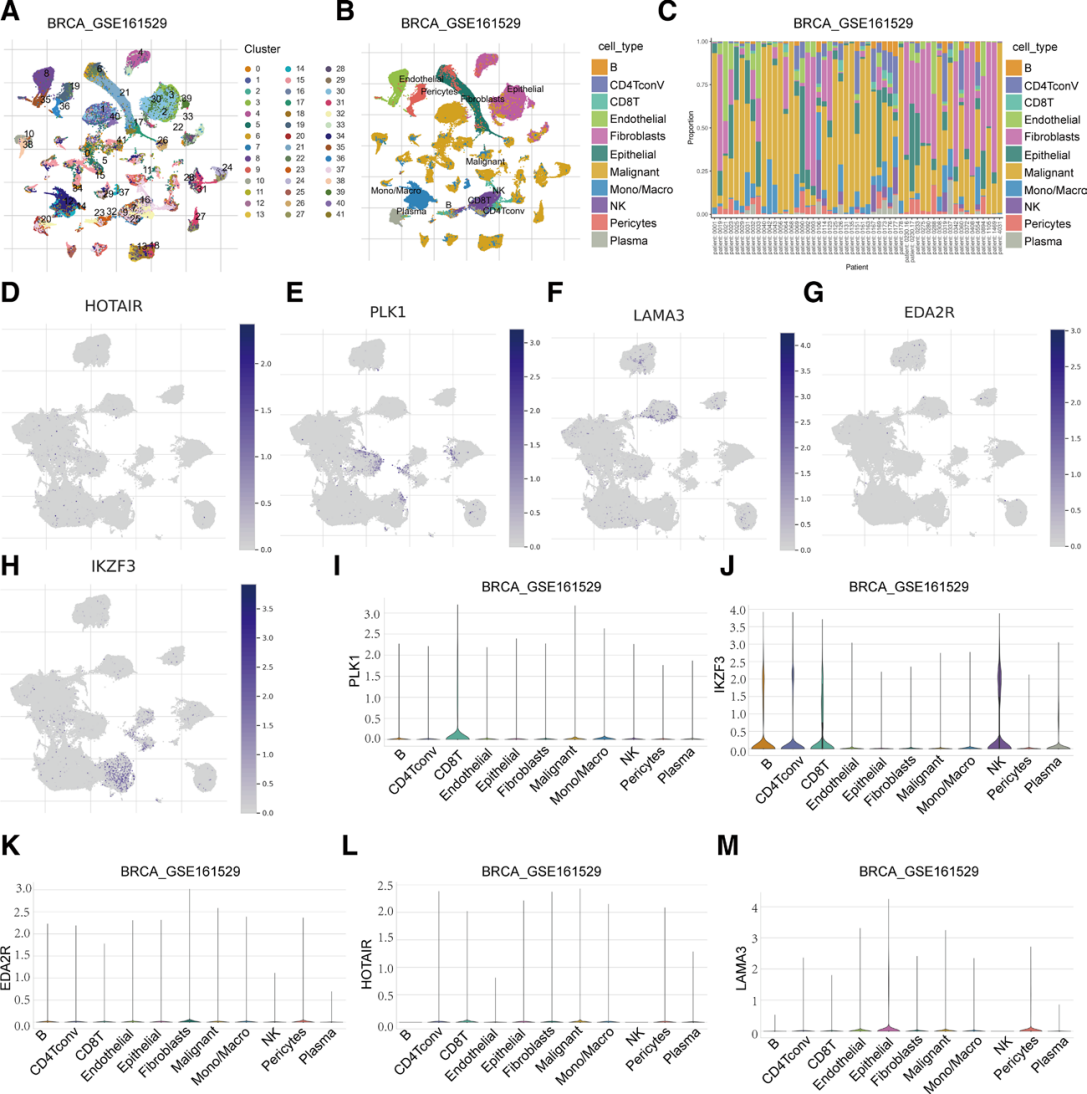
Correlation between ANRGs and the tumor microenvironment in single-cell sequence data. (A–C) Classification and statistics of cell types in the BRCA_GSE161259 data set. (D–M) Distribution and expression of the 5 ANRGs (PLK1, IKZF3, ED2AR, HOTA1R, and LAMA3). ANRGS = anoikis-related genes.

Using the BRCA_GSE161529 dataset from the TISCH database, we analyzed the expression of 5 ANRGs in single-cell RNA sequence data. Dataset GSE161529 revealed 41 cell clusters and 11 average cell types. The distribution and number of different cell categories (Fig. 8A–C). Our findings indicated that PLK1 and HOTAIR were predominantly expressed in both malignant cells and immune cells (CD8T and mononuclear/macrophage; Fig. 8D, E, I, and L). Additionally, IKZF3 was predominantly expressed in immune cells (B cells, CD4+ conventional T cells, CD8+ T cells, natural killer cells; Fig. 8H and J). EDA2R was mainly expressed in stromal cells (fibroblasts and pericytes; Fig. 8G and K), whereas LAMA3 was predominantly expressed in stromal cells (epithelial cells and pericytes; Fig. 8F and M).

### 3.9. Kaplan–Meier plot of EDA2R for pan-cancer

**Figure 9.**
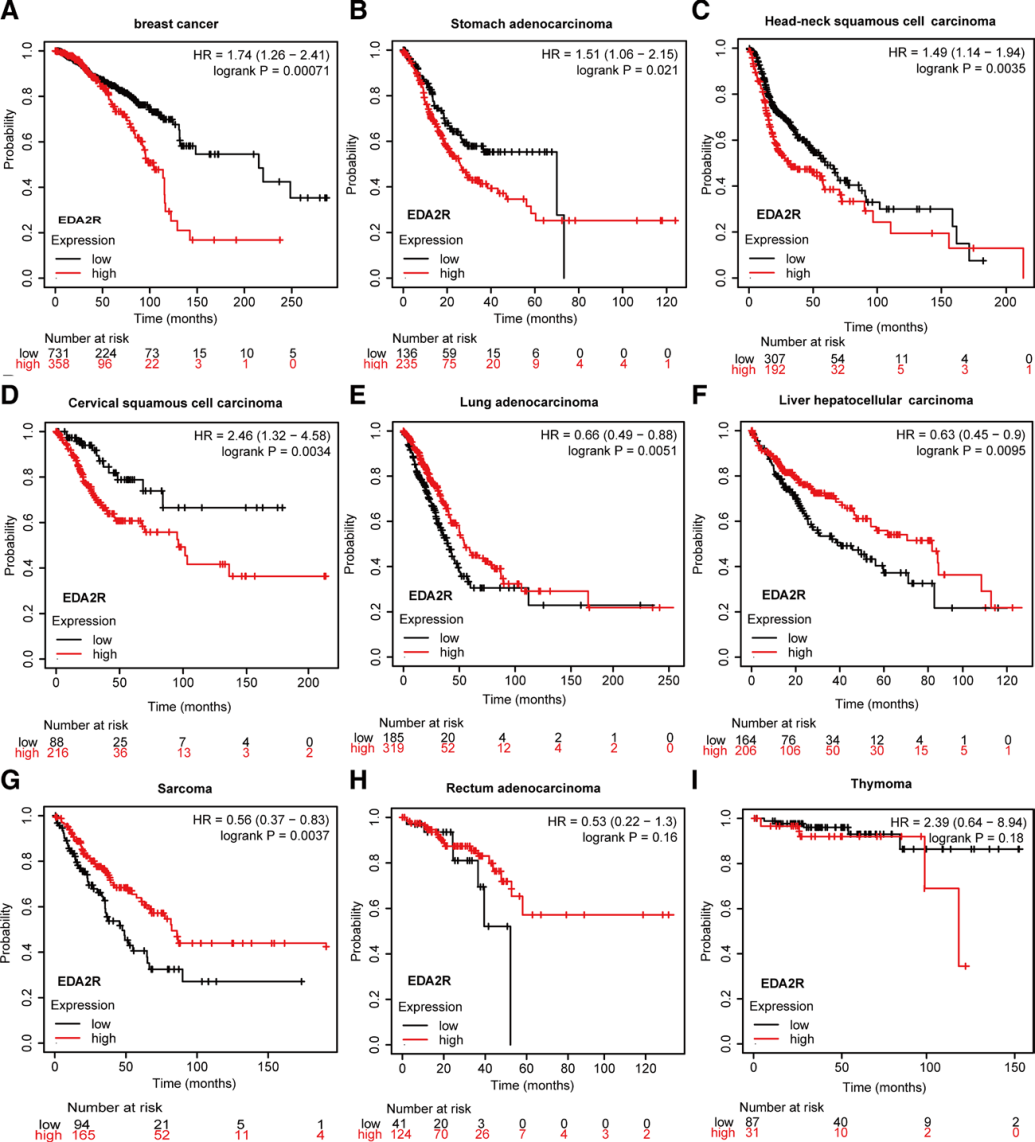
(A–I) Relationship between EDA2R transcriptional expression and prognosis in 9 types of tumors (breast cancer, stomach adenocarcinoma, head and neck squamous cell carcinoma, cervical squamous cell carcinoma, lung adenocarcinoma, liver hepatocellular carcinoma, sarcoma, rectum adenocarcinoma, and thymoma).

The relationship between EDA2R transcription and prognosis in various tumor types was examined using the KM Plotter database. Survival curves revealed that higher levels of EDA2R transcription were linked to poorer overall survival in breast cancer, stomach adenocarcinoma, head and neck squamous cell carcinoma, and cervical squamous cell carcinoma (Fig. 9A–D). However, patients with increased EDA2R expression exhibited a better prognosis in lung adenocarcinoma, liver hepatocellular carcinoma, and sarcoma (Fig. 9E–G). Notably, there was no association between EDA2R expression and prognosis in rectal adenocarcinomas and thymomas (Fig. 9H and I).

## 4. Discussion

Breast cancer exhibits significant heterogeneity, leading to variations in its biological behavior and clinical prognosis.^[15]^ Prognostic factors, such as estrogen receptor or progesterone receptor status, HER2 status, Ki-67, tumor size, and metastasis to axillary lymph nodes play a crucial role in breast cancer. Additionally, the molecular subtype of breast cancer is closely linked to personalized treatment choices and patient prognosis.^[16]^ However, the mechanisms underlying the progression of breast cancer remain unclear. Existing models lack widespread acceptance owing to their limited validation. Therefore, there is an urgent need to establish robust prognostic markers to improve the prognosis of breast cancer patients.

Anoikis is a specific apoptosis mechanism in which normal epithelial or endothelial cells adhere to the ECM through specific integrins. Activation of the anoikis pathway occurs when cells are detached from the ECM, and anoikis can be avoided when cells are inoculated with anti-integrin antibodies.^[17]^ The mechanism of resistance to anoikis remains elusive; however, some studies have reported that septin is recruited by blebs and interacts with the oncogenic driver NRAS to promote the activation of tumor growth signals. Bleb formation promotes the long-term survival of cells detached from the ECM and increases tumor metastasis and chemoresistance.^[18]^ Anoikis has been implicated in various biological behaviors of tumors; hence, the ANRG signature can be used as a marker for tumor prognosis.

Based on the TCGA breast cancer dataset, we identified 142 differentially expressed ANRGs. Sixteen ANRGs associated with prognosis were identified using univariate Cox regression analysis. ANRG signature risk scores were established using multivariate Cox regression and LASSO regression analyses. Finally, we developed and validated a 5-gene model capable of forecasting the outcomes of patients with breast cancer. This study employed the ANRGs signature risk score to classify breast cancer patients into high- and low-risk groups, enabling the identification of noticeable differences in immune cell infiltration, particularly CD8 T cells, between these subgroups. The composition of immune-infiltrating cells has been linked to the prognosis of patients with tumors. Using a single-cell database, we analyzed the expression levels of the 5 genes in different cells within the tumor microenvironment. The findings of this analysis provide valuable insights into the advancement of tumor immunotherapy.

We developed an ANRG signature that demonstrated a strong correlation with survival rates of patients with breast cancer. This signature consists of 5 genes (HOTAIR, PLK1, LAMA3, EDA2R, and IKZF3) that are closely linked to tumor development. HOTAIR overexpression in epithelial cancer cells causes genome-wide re-targeting of polycomb repressive complex 2 (PRC2), resulting in changes to lysine 27 methylation of histone H3. This alteration leads to increased cancer invasiveness and metastasis in a PRC2-dependent manner.^[19]^ HOTAIR plays a role in regulating the expression and activation of c-Met and its membrane colocalization partner, Caveolin-1. This regulation occurs in response to a non-adhesive environment, allowing cells to evade anoikis.^[20]^ NF-κB subunit RelA facilitates the survival of detached esophageal squamous cell carcinoma (ESCC) cells by preventing anoikis through the PLK1/β-catenin pathway.^[21]^ Patients with estrogen receptor-positive (ER+)/human epidermal growth factor receptor 2-negative (HER2-) breast cancer, who exhibit high expression of polo-like kinase 1 (PLK1), experience poorer median progression-free survival and are more susceptible to resistance against palbociclib.^[22]^ The frequencies of LAMA3 methylation and the average chain methylation index in tumors were significantly higher than those in normal tissues. Additionally, there is a correlation between the frequency of LAMA3 promoter methylation in breast tumors and an increase in tumor size and stage.^[23]^ In our study, upregulated EDA2R expression was accompanied by poorer prognosis in patients with breast cancer; nevertheless, additional research demonstrated that EDA2R is implicated in P53-mediated anoikis in tumor cells.^[24]^ To address this contradiction, we conducted a thorough analysis using the KM Plotter database to investigate the association between EDA2R expression and prognosis in various cancers. The results revealed that elevated EDA2R expression was associated with lower overall survival in patients with breast cancer. In this study, we observed that EDA2R expression was correlated with worse outcomes in both ANRG models, namely, consistency clustering (Figs. 2C and 3A) and ARG risk score (Fig. 6B). This finding aligns with the results obtained from the KM Plotter database analysis. Through our analysis of the tumor microenvironment, we made an intriguing discovery regarding the relationship between EDA2R expression and the proportion of immune-infiltrating cells. We found that EDA2R expression was positively associated with the proportion of immune-infiltrating cells, such as M2 macrophages and resting mast cells. In contrast, there was a negative correlation between EDA2R expression and the proportion of activated CD4+, CD8+, and follicular helper T cells (Fig. 5E). In the BRCA_GSE161529 dataset from the TISCH database, we observed that EDA2R was predominantly expressed in stromal cells, such as fibroblasts and pericytes (Fig. 8G and K). Based on our findings, EDA2R may have an impact on tumor prognosis through its influence on the tumor microenvironment. Additionally, our study demonstrated that knocking down IKZF3 in HER2-specific chimeric antigen receptor T cells, which target breast cancer cells, resulted in the enhanced killing of cancer cells in both in vitro and xenograft models. This enhancement can be attributed to the activation and proliferation of T-cells.^[25]^ IKZF3 overexpression can lead to a decrease in PRDM1 expression, which is associated with resistance to anoikis and poorer prognosis in individuals diagnosed with lung cancer.^[26]^ Feng et al developed a prognostic algorithm for breast cancer by utilizing the chromatin regulator-related gene signature and found that IKZF3 had a positive impact on overall survival, which is consistent with the findings of our study.^[27]^

The tumor microenvironment consists of not only malignant cells but also a diverse range of stromal and immune infiltrating cells, which play a crucial role in promoting tumor proliferation, development, metastasis, and immune evasion.^[28]^ The density, composition, functional status, and tissue-specific immune architecture of tumor leukocyte infiltration can provide valuable information for assessing overall survival and predicting chemotherapeutic sensitivity.^[29]^ We assessed the distribution of 22 different types of immune cells among different risk subgroups. Notably, in the subgroup with a higher risk score and poorer survival, there was a significant decrease in CD8+ T cell infiltration, indicating its significant role in breast cancer progression. Furthermore, we observed an inverse correlation between the proportion of CD8+ T cells and activated CD4+ memory T cells and the ANRG risk score, while M2 macrophages showed a positive correlation with the risk score. Metha et al reported that TAMs interfere with the tumor immune cycle by restricting antigen presentation and decreasing the response of cytotoxic T lymphocytes while boosting malignant cell longevity, angioplasty, and metastasis.^[30]^ This finding indicates that an immunosuppressive microenvironment may contribute to worse outcomes in patients with breast cancer who have high ANRGs signature risk scores. The reduced sensitivity to standard chemotherapy drugs in such patients may have contributed to their poor prognosis.

Despite the excellent performance of the ARG signature and nomogram in predicting the prognosis of breast cancer patients, it is important to acknowledge a major limitation of this study. All breast cancer specimens and information used in this study were obtained from publicly available databases. Therefore, it is crucial to conduct future studies to validate these results using clinical data.

In conclusion, our study demonstrated that the 5-gene model we developed is a reliable indicator of survival in patients with breast cancer. Furthermore, nomograms based on this signature of ANRGs can be effectively utilized to generate personalized therapies for breast cancer in clinical practice. Therefore, the ANRG signature and nomogram established in this study hold great potential for guiding clinical decisions regarding the treatment of patients with breast cancer.

## Author contributions

**Conceptualization:** Chaoyi Tang, Jiehua Li.

**Data curation:** Chaoyi Tang, Jiehua Li.

**Formal analysis:** Jiehua Li.

**Funding acquisition:** Jiehua Li.

**Investigation:** Jiehua Li.

**Methodology:** Chaoyi Tang, Jiehua Li.

**Project administration:** Liuqing Qin, Jiehua Li.

**Software:** Chaoyi Tang.

**Validation:** Liuqing Qin.

**Writing – original draft:** Chaoyi Tang.

**Writing – review & editing:** Jiehua Li.

## Supplementary Material




